# The impact of penicillin allergy labels on antibiotic and health care use in primary care: a retrospective cohort study

**DOI:** 10.1186/s13601-017-0154-y

**Published:** 2017-06-07

**Authors:** Tanly Su, Berna D. L. Broekhuizen, Theo J. M. Verheij, Heike Rockmann

**Affiliations:** 1 Julius Center for Health Sciences and Primary Care, University Medical Center Utrecht, Utrecht, The Netherlands; 2 Department of Dermatology and Allergology, University Medical Center Utrecht, G02.129, P.O. Box 85500, 3508 GA Utrecht, The Netherlands

**Keywords:** Allergy registration, Penicillin allergy, Drug hypersensitivity, Antimicrobial stewardship, Primary care

## Abstract

**Background:**

Suspected penicillin allergy (Pen-A) is often not verified by diagnostic testing. In third line penicillin allergy labels were associated with prescription of broad spectrum antibiotics, hospital stay duration and readmission.

**Objective:**

Assess the impact of Pen-A labels on antibiotic and health care use in primary care.

**Methods:**

A retrospective cohort study was conducted in primary care in the Utrecht area, the Netherlands. All patients registered with a penicillin allergy on 31 December 2013 were selected from the General Practitioner Network database. Each patient with a Pen-A label was matched for age, gender, follow-up period with three patients without Pen-A label. Risk (OR) of receiving a reserve and second choice antibiotic, number and type of antibiotics prescribed during follow-up and number of GP contacts were compared between the two cohorts.

**Results:**

Of 196,440 patients, 1254 patients (0.6%) with a Pen-A label were identified and matched with 3756 patients without Pen-A label. Pen-A labels resulted in higher risk of receiving ≥1 antibiotic prescription per year (OR 2.56, 95% CI 2.05–3.20), ≥1 s choice antibiotic prescription per year (OR 2.21 95% CI 1.11–4.40), and ≥4 GP contacts per year (OR 1.71 95% CI 1.46–2.00). The chance of receiving tetracyclins (OR 2.24, 95% CI 1.29–3.89), macrolides/lincosamides/streptogamins (OR 8.69, 95% CI 4.26–17.73) and quinolones (OR 2.59, 95% CI 1.22–5.48) was higher in Pen-A patients.

**Conclusions:**

In primary health care Pen-A labels are associated with increased antibiotic use, including second choice antibiotics, and more health care use.

**Electronic supplementary material:**

The online version of this article (doi:10.1186/s13601-017-0154-y) contains supplementary material, which is available to authorized users.

## Background

Penicillins are the most prescribed antibiotics worldwide [1–5] due to their high effectiveness, low cost and minimal side effects [6]. Unfortunately, penicillins are also a frequent cause of suspicion of antibiotic allergies [1, 2]. Prevalences of physician or patient reported suspected penicillin allergy (Pen-A) varying from 2 to 16% were reported, however the true prevalence in the general population is unknown, and remains difficult to determine due to varying study populations and study designs [7, 8].

Diagnostic workup for Pen-A includes detailed patient history, skin testing, in vitro testing and oral drug challenge. In Dutch primary care guidelines drug allergy testing is not addressed. Diagnostic workup takes place in specialized academic centers following European Academy of Allergy and Clinical Immunology (EAACI) guidelines [9]. Studies on outpatient adults in secondary and tertiary care showed that an alleged Pen-A can only be confirmed in the minority (16.5–29.0%) of patients [10]. Possible explanations for over reporting of Pen-A might be that non immunological side effects or disease symptoms such as viral exanthema are alleged to drug allergy. Studies in tertiary care showed that Pen-A labeling in patient charts is associated with more use of broad-spectrum antibiotics, particularly macrolides, quinolones, tetracyclin [11] and vancomycin [12, 13]. Use of these reserve antibiotics have been associated with higher risk of treatment failure, adverse drug reactions, complications as Clostridium difficile, MRSA and vancomycin-resistant Enterococcus infections, higher costs and it reinforces the development of antibiotic resistance [11]. Furthermore, Pen-A patients showed longer duration of hospitalization and more readmissions than non-Pen-A patients [11–13]. Another study in the United States also reported a higher mortality during admission in patients with a Pen-A label [13]. One single recent study conducted in a primary care population in the United States [n = 232,616 patients; 199 general practioners (GPs)] also revealed, that patients with Pen-A documentation received more fluoroquinolones, clindamycin and vancomycin [14].

The impact of Pen-A labels in primary care is however still poorly investigated, while the majority (80%) of antibiotics are prescribed in primary health care [15] and most allergy labels are probably assigned in primary care. The aim of this study was to assess the impact of Pen-A registration in primary care on health care related factors such as the number and type of antibiotics prescribed, second choice antibiotic prescriptions and GP contacts.

## Methods

### Setting and study population

This study was conducted in an extended primary care population in the Utrecht area of The Netherlands using data from the Utrecht General Practitioners Network (UGPN) database [16].

The UGPN database comprises anonymous routine healthcare data from electronic medical records extracted from general practices sharing their data with the UMC Utrecht. Our study extracted data from the electronic medical records of approximately 45 general practices involving 80 GPs and 196,440 patients enlisted in 2014 [16]. The study population was regarded as a representative sample of the Dutch population [16]. All the GPs had been properly trained in using the International Classification of Primary Care (ICPC) coding system and had an average of 10 years of experience [17].

The design of this study was a retrospective matched cohort study. Patients with and without the risk factor “Pen-A” were selected and observed over time for the outcomes [antibiotic (AB) use or GP use].

### Patient selection

All patients registered with a registration of “drug allergy” (ICPC A85.01) or “allergy” (ICPC code A12) on key date 31 December 2013 were extracted from the UGPN-database along with data concerning age, gender, atopic comorbidity (asthma, allergic rhinitis, eczema, allergic conjunctivitis), antibiotic prescription indications (by ICPC-codes), all antibiotic prescriptions and GP contacts. Data describe the period from February 1920 to July 2014. Data extraction was performed in November 2015. The accompanying free text of allergy description was checked to identify penicillin allergic patients. Penicillin was defined as all class ATC-J01C penicillin beta-lactam antibiotics including ampicillin and amoxicillin amongst others. Registration of Pen-A was defined as registration of the respective ICPC-code and description of penicillin beta-lactam antibiotics combined with the terms allergy, hypersensitivity or typical allergy symptoms (urticaria, angio-edema, rash, anaphylaxis). Information about specific symptoms and time relations was not adequate enough to distinguish reliably between immediate and non-immediate allergy. Patients without this specific registration were excluded. Each selected patient with a Pen-A label was matched to three patients without this label from the UGPN-database based on age, sex, GP practice and duration of follow-up after date of allergy registration.

This study was judged by the local Medical Ethical Review Committee as non-Medical Research Involving Human Subjects Act research project (reference number WAG/om/15/032309).

### Collected data classification

The primary outcome measure of this study was antibiotics prescribed during follow-up including second choice antibiotics. Prescribed antibiotics were grouped into the following ten Anatomical Therapeutic Chemical (ATC) classes: tetracyclins (J01A); amphenicols (J01B); beta-lactam antibacterials, penicillins (J01C); other beta-lactam antibacterials (J01D); sulfonamides and trimethoprim (J01E); macrolides, lincosamides and streptogramins (J01F); aminoglycoside antibacterials (F01G); quinolone antibacterials (J01M); combinations of antibacterials (J01R); other antibacterials (J01X) [31]. Second choice antibiotics were defined as antibiotics different from the recommended antibiotics for the specific indications by the Dutch guidelines for primary care [18–22]. To do so, the specific antibiotic prescription was linked to the coded diagnosis for which the antibiotic was prescribed. For bronchial infection, pharyngotonsillitis, otitis media acuta, acute rhinosinusitis and bacterial skin infections, narrow-spectrum beta-lactams (amoxicillin, penicillin) are listed as first choice of treatment in primary care [18–22]. All other antibiotics were considered second choice antibiotics for the respective diagnosis. As secondary outcome, number of GP contacts per year during follow-up (after beta-lactam antibiotic allergy registration) was evaluated.

### Statistical analysis

Descriptive analysis was used to determine the prevalence of beta-lactam allergy labels and patient characteristics of both groups. Patient characteristics at baseline were compared between the two groups using the Mann–Whitney U test and the independent samples t-test for the continuous variables. For the categorical variables the chi^2^-test or the Fisher’s exact test was used. P-values <0.05 were considered statistically significant. Patient year was used as unit of analyses, outcomes were expressed as the total number of events divided by the total number of patients times the duration of follow-up in years as denominator.

The association between beta-lactam antibiotic allergy labels and the defined outcomes was analyzed with logistic regression and presented in odds ratios (OR) with 95% confidence intervals (CI). Continuous outcomes were analyzed with linear regression and expressed in regression coefficients (B) with 95% CI. Analyses were adjusted for age, gender, atopic comorbidities (asthma, allergic rhinitis, eczema, allergic conjunctivitis, allergy other than for penicillin), GP practice and number of GP contacts.

Analyses were repeated in the subgroups females, antibiotic users and those with atopic comorbidities. All statistical analyses were conducted with SPSS, version 21 (SPSS, Inc., Chicago, IL).

## Results

Out of the database of 196,440 enlisted patients [52% female; mean (SD) age 38 (22) years] in 2014, 1254 (0.6%) individual patients [66.9% females; mean (SD) age 47 (26) years] with a Pen-A registration were identified with a median duration of follow-up of 4.5 years (IQR 5 years). All patients could be matched with three patients without Pen-A registration resulting in a total study population of 5010 patients. Date of Pen-A registration ranged from 24 November 1989 to 17 February 2014. Atopic comorbidities were significantly more often registered in Pen-A patients compared to non-Pen-A patients (P < 0.05). The most common indications for antibiotic prescription were respiratory (27.5%) and urinary (28.4%) disease followed by skin infections (8.0%) and ear infections (3.6%) without significant difference between both groups (Additional file 1: Table S1). Data concerning indication for antibiotic prescription were missing in 13.9% of the prescriptions (14.2% in the Pen-A group; 13.8% in non-Pen-A group). All other data were complete. For both Pen-A and non-Pen-A patients tetracyclins were the most prescribed second choice antibiotic for respiratory infections. For skin infections it were ‘macrolides, lincosamides and streptogamins’. For ear infections ‘macrolides, lincosamides and streptogamins’ were the most prescribed second choice antibiotic for Pen-A patients, and sulfonamides for non-Pen-A patients (Additional file 2: Table S2a, Additional file 3: Table S2b, Additional file 4: Table S2c).

### Antibiotic use

Antibiotic and primary health care use per year is shown in Table 1. Out of the 5010 included patients, 497 patients (9.9%) received at least one antibiotic prescription per patient year. Pen-A patients received more than twice as often (19.0%) an antibiotic prescription (number of prescriptions: mean 0.8; SD 0.4) than non-Pen-A patients (6.9%) (number of prescriptions: mean 0.3; SD 0.9). Pen-A patients more often received at least one antibiotic prescription per year compared to non-Pen-A patients (adjusted OR 2.56, 95% CI 2.046–3.197). Regression analysis showed that Pen-A patients received 0.39 antibiotic prescription more per year (95% CI 0.183–0.600) (Table 2).

**Table 1:** Patients antibiotic prescriptions and GP contacts in primary care per patient year (relative effect)

Outcome	Total n = 5010	Pen-A patients n = 1254	Non-Pen-A patients n = 3756	Unadjusted OR	95% CI	Adjusted OR	95% CI
No. of patients treated with							
≥1 AB prescription per patient year^c,e^	497 (9.9%)	238 (19.0%)	259 (6.9%)	*3.16*	*2.617*–*3.822*	*2.56* ^*a*^	*2.046*–*3.197*
≥1 2nd choice AB prescriptions per patient year^d,e^	47 (0.9%)	18 (1.4%)	29 (0.8%)	*1.26*	*1.008*–*1.582*	*2.21* ^*a*^	*1.107*–*4.402*
≥1 Tetracyclins^e^	68 (1.4%)	35 (2.8%)	33 (0.9%)	*3.24*	*2.004*–*5.235*	*2.24*	*1.287*–*3.887*
≥1 Beta-lactam, penicillins^e^	101 (2.0%)	30 (2.4%)	71 (1.9%)	1.27	0.826–1.959	0.80	0.482–1.342
≥1 Beta-lactam, others^e^	2 (0.0%)	1 (0.1%)	1 (0.0%)	3.00	0.187–47.947	6.09	0.360–103.149
≥1 Sulfonamides and trimethoprim^e^	32 (0.6%)	18 (1.4%)	14 (0.4%)	*3.89*	*1.930*–*7.849*	2.07	0.908–4.724
≥1 Macrolides, lincosamides and streptogramins^e^	64 (1.3%)	53 (4.2%)	11 (0.3%)	*15.02*	*7.823*–*28.855*	*8.69*	*4.261*–*17.728*
≥1 Quinolones^e^	34 (0.7%)	15 (1.2%)	19 (0.5%)	*2.38*	*1.206*–*4.700*	*2.59*	*1.221*–*5.479*
≥1 Other antibacterials^e^	82 (1.6%)	30 (2.4%)	52 (1.4%)	*1.75*	*1.109*–*2.749*	1.31	0.767–2.231
No. of patients with							
≥4 GP contacts per patient year^c,e^	2353 (47.0%)	761 (60.7%)	1592 (42.4%)	*2.10*	*1.845*–*2.396*	*1.71* ^*b*^	*1.460*–*1.999*

*Pen*-*A* penicillin allergy label, *No.* number, *AB* antibiotic, *GP* general practitioner, *OR* odds ratio, *CI* confidence interval

Italic values represent significant differences between Pen-A and non-Pen-A

^a^adjusted for ≥4 GP-contacts per year, gender, age >12 years, comorbidities (R96, R97, S87, F71, A12), GP practice

^b^Adjusted for gender, age ≥12, comorbidities (R96, R97, S87, F71, A12), GP practice

^c^Based on the median per year

^d^2nd choice AB based on Dutch guidelines for primary care. Data obtained by matching AB prescriptions with corresponding ICPC-codes

^e^Per patient year = total number of events/(total number of patients × total years of follow-up)

**Table 2:** Total number of antibiotic prescriptions and GP contacts in primary care per patient year (absolute effect)

Outcomes	Total n = 5010	Pen-A patients n = 1254	Non-Pen-A patients n = 3756	Unadjusted B	95% CI	Adjusted B^a^	95% CI
No. of AB per PY (%)^d^	2110 (100.0%)	1051 (100.0%)	1059 (100.0%)	*0.556*	*0.377 to 0.736*	*0.392*	*0.183 to 0.600*
No. of 2nd choice AB per PY^b^ (%)^d^	269.4 (5.4%)	93.7 (7.5%)	175.7 (4.7%)	0.039	−0.005 to 0.084	*0.024*	*0.008 to 0.111*
Various types of antibiotics: No. of AB per PY(%)^d^							
Tetracyclins	332 (15.7%)	136 (12.9%)	195 (18.4%)	*0.057*	*0.036 to 0.078*	*0.029*	*0.005 to 0.053*
BL, penicillins	501 (23.7%)	142 (13.5%)	372 (35.1%)	0.014	−0.008 to 0.037	−0.023	−0.049 to 0.002
BL, others	21 (1.0%)	7 (0.7%)	14 (1.3%)	0.002	−0.010 to 0.013	0.004	−0.010 to 0.018
Sulfonamides/trimethoprim	140 (6.6%)	70 (6.7%)	71 (6.7%)	*0.037*	*0.022 to 0.051*	*0.027*	*0.010 to 0.043*
Macrolides^c^	537 (25.4%)	458 (43.6%)	78 (7.4%)	*0.345*	*0.176 to 0.514*	*0.263*	*0.066 to 0.60*
Quinolones	195 (9.2%)	78 (7.4%)	116 (11.0%)	*0.032*	*0.000 to 0.063*	0.034	−0.002 to 0.007
Other antibacterials	373 (17.7%)	159 (15.1%)	214 (20.2%)	*0.070*	*0.045 to 0.096*	*0.058*	*0.029 to 0.087*
No. of GP contacts per PY	6.3	16.0	3.0	*3.732*	*3.089 to 4.375*	*3.052*	*2.316 to 3.788*

*Pen*-*A* penicillin allergy label, *B* regression coefficient per patient year, *No.* number, *AB* antibiotic prescriptions, *PY* patient year, *BL* beta-lactam, *GP* general practitioner, *OR* odds ratio, *CI* confidence interval

Italic values represent significant differences between Pen-A and non-Pen-A

^a^Adjusted for gender, age >12 years, comorbidities (R96, R97, S87, F71, A12), GP practice, ≥4 GP contacts/year

^b^2nd choice AB based on Dutch guidelines for primary care. Data obtained by matching AB prescriptions with corresponding ICPC-codes

^c^Lincosamides and streptogramins included

^d^Percentage of total number of AB prescriptions per year

Prescription of second choice antibiotics per year occurred more often to Pen-A patients (adjusted OR 2.21, 95% CI 1.107–4.402) (Table 1) resulting in 0.02 more prescriptions of second choice antibiotics more per year in Pen-A patients compared to non-Pen-A patients (95% CI 0.008–0.111). After adjustment for overall number of prescriptions, Pen-A patients more often received second choice antibiotics, in particular tetracyclins (adjusted OR 2.24, 95% CI 1.287–3.887), quinolones (adjusted OR 2.59, 95% CI 1.221–5.479), and macrolides, lincosamides and streptogamins (adjusted OR 8.69, 95% CI 4.261-17.728), than non-Pen-A patients. This is seen over all studied indications. Both in patients with and without Pen-A, in sinusitis and pneumonia, the percentage of secondary choice antibiotics was high (Additional file 3: Table S2b, Additional file 4: Table S2c). There was no difference in the number of penicillin prescriptions between the two groups (adjusted B −0.02, 95% CI −0.049 to 0.002).

The ranking and percentages of frequency of specific antibiotic prescriptions differed between the two groups (Table 3).

**Table 3:** Top 7 antibiotic groups used by the registered Pen-A and non-Pen-A patients during follow-up

	Registered Pen-A patients (n = 1254)	Non-Pen-A patients (n = 3756)
1	Macrolides: N = 850 (25.2%)	Beta-lactam, penicillins: N = 1733 (33.9%)
2	Tetracyclins: N = 677 (20.1%)	Other antibacterials: N = 1087 (21.2%)
3	Other antibacterials: N = 602 (17.8%)	Tetracyclins: N = 973 (19.0%)
4	Beta-lactam, penicillins: N = 509 (15.1%)	Quinolones N = 511 (10.0%)
5	Sulfanomides and trimethoprim: N = 397 (11.8%)	Sulfanomides and trimethoprim: N = 379 (7.4%)
6	Quinolones N = 325 (9.6%)	Macrolides: N = 379 (7.4%)
7	Beta-lactam, others: N = 16 (0.5%)	Beta-lactam, others: N = 55 (1.1%)

Multiple prescriptions of the same antibiotic during hospitalization were counted only once

Other antibacterial: ATC class J01X (Glycopeptide, Polymyxin, Imidazol derivates, nitrofuran derivates ect.)

*Pen*-*A* penicillin allergy label

### Primary health care use

There were 20,069 GP contacts per year in the Pen-A group and 11,377 contacts in the non-Pen-A group. In the total study population of 5010 patients the median number of GP contacts per year was 3.7 (SD 6.1). Pen-A patients had significantly more GP contacts per year compared to non-Pen-A patients [median (SD) 5.5 (8.3) vs. 3.3 (5.4) P < 0.05]. A Pen-A label increased the odds of having ≥4 GP-contacts (based on the median GP contact in the normal population) per patient year (adjusted OR 1.71 95% CI 1.460–1.999) (Table 2).

## Discussion

This is the first study in Europe investigating the impact of a Pen-A registration in primary care. It shows that a Pen-A label is related to a higher use of antibiotics (19% of Pen-A patients received an antibiotic prescription per year compared to 6.9% of non-Pen-A patients), in particular regarding reserve and second choice antibiotics. Furthermore, patients with registration of Pen-A use more primary health care. Remarkably, in contrast to previous studies [11, 14, 23] Pen-A patients did not receive less prescriptions of penicillins compared to non-Pen-A patients in our study. Possible explanation could be that prescribing GPs in our study were aware of the overestimation of Pen-A labeling and consciously prescribed penicillins to Pen-A patients based on their own experience.

Before drawing conclusions from our results, potential limitations should be considered. Although patients with and without Pen-A registration were matched on age, sex, GP practice and follow-up period, results in our study may be explained by residual confounding mainly related to unmeasured comorbidity. We did not have any information concerning comorbidities, antibiotic and health care use before the moment of Pen-A registration at our disposal. Pen-A patients may have more comorbidities, resulting in more antibiotic use and health care use compared to non-Pen-A patients to begin with. This certainly has to be kept in mind when interpreting the results of our study. Another limitation is that our data did not include information about the severity of the allergy reactions or differentiation between immediate and non-immediate allergy.

Although our study did not aim to assess the prevalence of Pen-A labels in primary care, the prevalence of 0.6% is considerably lower than previously reported in the literature (2.0–15.6%) [7, 8, 14, 24]. A recent mono center study in Dutch primary care also showed a very low prevalence of 2.0%, possibly implicating country specific differences [7]. A recent study in an adult primary care population in the United States by Shah et al. [14] observed a high prevalence of 15.6%. This study, included 232,616 patients seen by 199 primary care providers, also showed a high variability in Pen-A documentation between the different primary care providers varying from 7.9 to 24.8% [14]. Therefore differences in reported prevalences might be explained by differences in study designs but also other factors such as the variation in Pen-A documentation between GPs. Stricter antibiotic allergy labeling behavior of the Dutch GPs and the low antibiotic prescribing rate in The Netherlands and in particular in the studied population might contribute to the low prevalence in our study, since a higher antibiotic prescription rate is known to be related to a higher frequency of (suspicion of) antibiotic allergy [12, 13, 25]. In our study only 9.9% of the total study population received at least one antibiotic prescription per patient year compared to 18.2% of the total Dutch population [3]. This might be caused by a superior motivation and attention on following the guidelines more strictly in GPs actively participating in our research network [26]. However, we have no reason to think that this lower prevalence of Pen-A labeling did influence the relation between labeling, antibiotic use and consultation rates.

Our data confirm the results of a recent study conducted in primary care population in the United States (n = 232,616; 199 GPs) reporting that patients with Pen-A registration were of higher risk to receive fluoroquinolones, clindamycin and vancomycin [14]. These reserve antibiotics are associated with higher costs, more adverse reactions and complications and a high risk of developing antimicrobial resistance [27, 28]. Increased usage of macrolides and clindamycin, quinolones and tetracyclin in patients with Pen-A registration was also shown in a recent study in Dutch tertiary care and tertiary care studies from Israel, the United States and the United Kingdom [4, 5, 11, 29]. These studies on hospitalized patients also showed an increased risk of longer duration of hospital admission and re-admission.

### Implications for research and practice

Our finding that patients with a penicillin allergy label use 0.4 more antibiotic prescription yearly may seem small on the total number of prescriptions. However, given the low prescription rate of antibiotics in the Netherlands (10.0 defined daily doses per 1000 inhabitants daily) compared to other European countries (e.g. France: 32.2 defined daily doses per 1000 inhabitants daily) or Unites States (US: 25 defined daily doses per 1000 inhabitants daily), [2, 4, 30–32] total effects are probably larger in high prescribing countries and with higher prevalences of penicillin allergy registration.

Our study shows that registration of penicillin allergy certainly affects treatment and health care use in primary care patients, but the effect is small in this low prescribing setting. However, considering the fact that most allergy labels are incorrect, the prescribing of beta-lactam antibiotics in our population was incorrectly avoided in most cases. Considering the emerge of antimicrobial resistance, efforts to decrease incorrect labeling are worthwhile [23].

Several studies in secondary or tertiary care have shown that active antibiotic allergy verification by skin testing and drug challenge decreases use of second choice antibiotics [10]. However, further studies are needed to investigate whether exclusion of Pen-A in primary care patients is cost-effective, and to quantify the impact on secondary and tertiary care (e.g. hospital admission, length of hospital admission, antimicrobial resistance) in order to draw up a national guideline for drug allergy testing in primary care.

 In conclusion, this study shows that a Pen-A label is related to more prescriptions of antibiotics, more prescriptions of second choice antibiotics and more GP contacts in primary care. Therefore improvement of Pen-A diagnosis and labeling could improve the rational use of antibiotics.

## Additional files

**Additional file 1: Table S1.** Indications for antibiotic prescriptions presented as ICPC-codes per tractus.

**Additional file 2: Table S2a.** Number of second choice antibiotic prescriptions per indication in primary care for total study population.

**Additional file 3: Table S2b.** Number of second choice antibiotic prescriptions per indication for Pen-A patients in primary care.

**Additional file 4: Table S2c.** Number of second choice antibiotic prescriptions per indication for non-Pen-A patients in primary care.

## Abbreviations

ATC: anatomical therapeutical chemical
B: regression coefficient
CI: confidence interval
GP: general practitioner
ICPC: International Classification of Primary Care
IQR: interquartile range
SD: standard deviation
OR: odds ratio
Pen-A: penicillin allergy
Non-Pen-A: non penicillin allergy
UGPN: Utrecht General Practitioners Network
WHO: World Health Organization
