# Supplementary material for: The impact of penicillin allergy labels on antibiotic and health care use in primary care: a retrospective cohort study
Source: Clin Transl Allergy. 2017 Jun 7;7:18. doi: 10.1186/s13601-017-0154-y (PMC5461748; doi:10.1186/s13601-017-0154-y)
Supplement: Supplementary file 1 — Additional file 1: Table S1. Indications for antibiotic prescriptions presented as ICPC-codes per tractus. [file 13601_2017_154_MOESM1_ESM.docx]

**Additional file 1: Table S1. Indications for antibiotic prescriptions presented as ICPC-codes per tractus**

| **Indications for AB prescriptions** | **Total** | **Pen-A** | | | **Non Pen-A** | |
| --- | --- | --- | --- | --- | --- | --- |
|  | n=5010 | Total n=1254 | | | Total n= 3756 | |
| **ICPC-codes*** | 8634 | 3385 | | | 5249 | |
| **Respiratory** | 2375 (27,5%) | 988 (29,2%) | | | 1387 (26,4%) | |
| **Ear** | 309 (3,6%) | 146 (4,3%) | | | 160 (3,0%) | |
| **Skin** | 695 (8,0%) | 285 (8,4%) | | | 410 (7,8%) | |
| **Urinary** | 2455 (28,4%) | 889 (26,3%) | | | 1566 (29,8%) | |
| Other | 1623 (18,8%) | 752 (22,2%) | | | 1003 (19,1%) | |
| *Missing* | 1177 (13,6%) | *479 (14,2%)* | | | *723 (13,8%)* | |
|  | | |  |  | |  |
| *AB= antibiotic, Pen-A= penicillin allergy label, ICPC=International Classification of Primary Care* *Multiple ICPC-codes per patient are possible *no difference between Pen-A and non Pen-A* | | | | | |  |
