# Supplementary material for: The impact of penicillin allergy labels on antibiotic and health care use in primary care: a retrospective cohort study
Source: Clin Transl Allergy. 2017 Jun 7;7:18. doi: 10.1186/s13601-017-0154-y (PMC5461748; doi:10.1186/s13601-017-0154-y)
Supplement: Supplementary file 2 — Additional file 2: Table S2a. Number of second choice antibiotic prescriptions per indication in primary care for total study population. [file 13601_2017_154_MOESM2_ESM.docx]

| **Additional file 2: Table S2a. Number of second choice antibiotic prescriptions per indication in primary care for total study population** | | | | | | | | | | | | | | | | | | | | |
| --- | --- | --- | --- | --- | --- | --- | --- | --- | --- | --- | --- | --- | --- | --- | --- | --- | --- | --- | --- | --- |
|  |  | **First choice*** | **Second choice*** | | |  | | |  | | |  | | |  | | |  |  |  |
| **Diagnosis (ICPC-code)** | **Total** | **BL, penicillins (%)** | **Total (%):** | ***Tetracyclins*** | ***BL, others*** | | ***Sulfonamides /trimethoprim*** | | | ***Macrolides***** | | | ***Quinolones*** | | | ***Other AB*** | | |  |  |
| **Respiratory** | 735 | **249 (33,9%)** | **486 (66,1%)** | 316 (43,0%) | 0 | | | 16 (2,2%) | | | 136 (18,5%) | | | 8 (1,1%) | | | 0 | |  |  |
| Acute/chronic sinusitis (R75) | 342 | **52 (15,2%)** | **290 (84,8%)** | 225 (65,8%) | 0 | | | 5 (1,5%) | | | 55 (16,1%) | | | 5 (1,5%) | | | 0 | |  |  |
| Acute tonsillitis (R76) | 137 | **103 (75,2%)** | **34 (24,8%)** | 3 (2,2%) | 0 | | | 2 (1,5%) | | | 29 (19,7%) | | | 0 | | | 0 | |  |  |
| Pneumonia (R81) | 256 | **94 (36,7%)** | **162 (63,3%)** | 88 (34,4%) | 0 | | | 9 (3,5%) | | | 62 (24,2%) | | | 3 (3,2%) | | | 0 | |  |  |
| **Ear** |  |  |  |  |  | | |  | | |  | | |  | | |  | |  |  |
| Acute otitis media (H71) | 213 | **127 (59,6%)** | **104 (40,4%)** | 5 (2,3%) | 0 | | | 16 (7,5%) | | | 83 (39,0%) | | | 0 | | | 0 | |  |  |
| **Skin** |  |  |  |  |  | | |  | | |  | | |  | | |  | |  |  |
| Other skin infections (S76) | 161 | **109 (37,7%)** | **52 (62,3%)** | 2 (1,2%) | 1 (0,6%) | | | 2 (1,2%) | | | 45 (28,0%) | | | 2 (1,2%) | | | 0 | |  |  |
|  |  |  |  |  |  | | |  | | |  | | |  | | |  | |  |  |
| *ICPC=International classification of Primary Care, BL= beta-lactam, AB=antibiotics*  *According to the Dutch Guidelines for Primary Care | | | | | | | | | | | | | | | | | | |  |  |

** inclusive lincosamides and streptogramins
