# Supplementary material for: The impact of penicillin allergy labels on antibiotic and health care use in primary care: a retrospective cohort study
Source: Clin Transl Allergy. 2017 Jun 7;7:18. doi: 10.1186/s13601-017-0154-y (PMC5461748; doi:10.1186/s13601-017-0154-y)
Supplement: Supplementary file 3 — Additional file 3: Table S2b. Number of second choice antibiotic prescriptions per indication for Pen-A patients in primary care. [file 13601_2017_154_MOESM3_ESM.docx]

**Additional file 3: Table S2b. Number of second choice antibiotic prescriptions per indication for Pen-A patients in primary care**

|  | | **First choice*** | **Second choice*** | |  | |  |  | | |  | |  | |
| --- | --- | --- | --- | --- | --- | --- | --- | --- | --- | --- | --- | --- | --- | --- |
| **ICPC-code**  **(Total)** | | **BL, penicillins (%)** | **Total (%):** | | ***Tetracyclins*** | | ***BL, others*** | ***Sulfonamides/ trimethoprim*** | | | ***Macrolides***** | | ***Quinolones*** | ***Other AB*** |
| **Respiratory**  **(203)** | | **53 (6,7%)** | **150 (18,9%)** | | 129 (16,2%) | | 0 | 12 (1,5%) | | | 96 (12,1%) | | 6 (0,8%) | 0 |
| Acute/chronic sinusitis  (135) | | **11 (8,1%)** | **124 (91,9%)** | | 89 (65,9%) | | 0 | 3 (2,2%) | | | 27 (20%) | | 5 (3,7%) | 0 |
| Acute tonsillitis  (50) | | **24 (48%)** | **26 (52,0%)** | | 2 (4,0%) | | 0 | 2 (4,0%) | | | 22 (44,0%) | | 0 | 0 |
| Pneumonia  (111) | | **18 (16,2%)** | **93 (83,8%)** | | 38 (34,2%) | | 0 | 7 (6,3%) | | | 47 (42,3%) | | 1 (0,9%) | 0 |
| **Ear** | |  |  | |  | |  |  | | |  | |  |  |
| Acute otitis media  (109) | | **25 (22,9%)** | **84 (77,1%)** | | 2 (1,8%) | | 0 | 5 (4,6) | | | 77 (70,6%) | | 0 | 0 |
| **Skin** | |  |  | |  | |  |  | | |  | |  |  |
| Other skin infections  (71) | | **30 (42,3%)** | **41 (57,7%)** | | 2 (2,8%) | | 1 (1,4%) | 0 | | | 37 (52,1%) | | 1 (1,4%) | 0 |
|  |  | |  |  | |  | | |  |  | |  |  |  |

*ICPC=International classification of Primary Care, BL= beta-lactam, AB=antibiotics*

*According to the Dutch Guidelines for Primary Care
** lincosamides and streptogramins included
