# Supplementary material for: The impact of penicillin allergy labels on antibiotic and health care use in primary care: a retrospective cohort study
Source: Clin Transl Allergy. 2017 Jun 7;7:18. doi: 10.1186/s13601-017-0154-y (PMC5461748; doi:10.1186/s13601-017-0154-y)
Supplement: Supplementary file 4 — Additional file 4: Table S2c. Number of second choice antibiotic prescriptions per indication for non-Pen-A patients in primary care. [file 13601_2017_154_MOESM4_ESM.docx]

**Additional file 4: Table S2c. Number of second choice antibiotic prescriptions per indication for non-Pen-A patients in primary care**

|  | **First choice*** | **Second choice*** | |  |  | |  | |  | |  | |
| --- | --- | --- | --- | --- | --- | --- | --- | --- | --- | --- | --- | --- |
| **ICPC-code (Total)** | **BL, penicillins (%)** | **Total (%):** | ***Tetracyclins*** | ***BL, others*** | | ***Sulfonamide/trimethoprim*** | | ***Macrolides***** | | ***Quinolones*** | | ***Other AB*** |
| **Respiratory (439)** | **243 (55,4%)** | **196 (44,6%)** | 187 (42,6%) | 0 | | 4 (9,1%) | | 50 (11,4%) | | 2 (4,6%) | | 0 |
| Acute/chronic sinusitis (207) | **41 (19,8%)** | **166 (80,2%)** | 136 (65,7%) | 0 | | 2 (9,7%) | | 28 (13,5%) | | 0 | | 0 |
| Acute tonsillitis (87) | **79 (90,8%)** | **8 (9,2%)** | 1 (1,1%) | 0 | | 0 | | 7 (8,0%) | | 0 | | 0 |
| Pneumonia (145) | **76 (52,4%)** | **69 (47,6%)** | 50 (34,5%) | 0 | | 2 (1,4%) | | 15 (10,3%) | | 2 (1,4%) | | 0 |
| **Ear** |  |  |  |  | |  | |  | |  | |  |
| Acute otitis media (122) | **102 (83,6%)** | **20 (16,4%)** | 3 (2,5%) | 0 | | 11 (9,0%) | | 6 (4,9%) | | 0 | | 0 |
| **Skin**  Other skin infections (90) | **79 (87,8%)** | **11 (12,2%)** | 0 | 0 | | 2 (2,2%) | | 8 (8,9%) | | 1 (1,1%) | | 0 |

*ICPC=International classification of Primary Care, BL= beta-lactam, AB=antibiotics*

*According to the Dutch Guidelines for Primary Care
** lincosamides and streptogramins included
